# A228 RISANKIZUMAB - DRUG INDUCED LIVER INJURY; A CASE REPORT

**DOI:** 10.1093/jcag/gwad061.228

**Published:** 2024-02-14

**Authors:** F Borahmah, B Salh, D Chahal

**Affiliations:** Department of Gastroenterology, The University of British Columbia, Vancouver, BC, Canada; Department of Gastroenterology, The University of British Columbia, Vancouver, BC, Canada; Department of Gastroenterology, The University of British Columbia, Vancouver, BC, Canada

## Abstract

**Background:**

Risankizumab is a humanized monoclonal antibody to IL-23. It was initially used in the treatment of plaque psoriasis with recent approval to extend use to treat moderate to severe Crohn’s disease with evidence of endoscopic healing and clinical remission of disease. In the drugs safety manual, it has been reported to have transient liver enzyme elevation that is asymptomatic and not to a degree requiring drug discontinuation. No reported data on steatohepatitis. In this article we describe a 28 year old female, who had no evidence of liver disease in the past who underwent treatment with Risankizumab and within several months developed evidence of steatohepatitis with radiological evidence of significant hepatomegaly.

**Aims:**

Case report details Drug induced liver injury to Risankizumab.

**Methods:**

Case Report

**Results:**

Drug induced liver injury with elevated liver enzyme levels and liver biopsy showing drug related liver changes likely in the setting of Risankizumab use.

**Conclusions:**

Risankizumab is a newly approved drug in the setting of Crohn's disease, although described in the drug assessment report that it may cause transient hepatitis or eleveted liver enzymes in the setting of lower drug dose for Psoriasis use, the higher drug level used in the setting of Crohn's disease has not been formally evaluated and no previous known case reports of Drug induced Liver Injury to Risankizumab causing severe steatohepatitis

**Funding Agencies:**

None

